# C-reactive protein–triglyceride–glucose index predicts acute pancreatitis severity: a retrospective cross-sectional study

**DOI:** 10.3389/fendo.2026.1964371

**Published:** 2026-09-21

**Authors:** Tiantian Xu, Yue Gao, Zewen Zhang, Lihui Lin, Zhen Ding

**Affiliations:** Department of Gastroenterology, The First Affiliated Hospital, Sun Yat-sen University, Guangzhou, China

**Keywords:** acute pancreatitis, C-reactive protein–triglyceride–glucose index, inflammation, insulin resistance, prognosis

## Abstract

**Objective:**

Early prediction of acute pancreatitis (AP) severity is essential for timely intervention and improved outcomes. The C-reactive protein–triglyceride–glucose index (CTI) is a simple, routine laboratory-derived biomarker. It integrates signals of inflammation and insulin resistance (IR) and has demonstrated predictive value in cardiovascular and metabolic diseases. However, its association with AP severity remains unknown.

**Methods:**

This retrospective cross-sectional study included AP patients admitted between January 2019 and January 2026. CTI was calculated as 0.412 × ln (CRP, mg/L) + ln [triglycerides (mg/dL) × fasting glucose (mg/dL)/2]. We assessed the association between CTI and AP severity using Spearman’s correlation, logistic regression, and receiver operating characteristic (ROC) curve analysis. Severity was graded according to both the revised Atlanta criteria (RAC) and the modified computed tomography severity index (MCTSI).

**Results:**

A total of 567 patients with AP were included in this study. The high CTI group had significantly more severe AP than the low CTI group under both the RAC and MCTSI grading systems (both *P* < 0.001). Spearman’s rank correlation confirmed a positive monotonic association (*P* < 0.001). In multivariate models, CTI independently predicted severity under the RAC (odds ratio [OR] 1.180, 95% confidence interval [CI] 1.027–1.357, *P* = 0.019) and MCTSI (OR 1.436, 95% CI 1.237–1.667, *P* < 0.001). For MCTSI ≥ 8, the area under the ROC curve (AUC) reached 0.763 (95% CI 0.681–0.845, *P* < 0.001). The combined model of CTI + white blood cell (WBC) + lactate dehydrogenase (LDH) yielded the highest AUCs across all endpoints (range 0.697–0.795).

**Conclusion:**

CTI independently predicts AP severity with moderate discriminative accuracy. Its routine availability and low cost support its potential as an adjunct for early risk stratification. Adding WBC and LDH further improved predictive performance, though the incremental benefit was modest.

## Introduction

Acute pancreatitis (AP) is an acute inflammatory disorder of the pancreas (1, 2), and its global incidence has risen steadily in recent years (3, 4). Approximately 20% of patients progress to moderate or severe acute pancreatitis (SAP) (5). Among those with severe disease, persistent organ failure is common and mortality rates reach 20–40% (5–7). Beyond its clinical severity, SAP imposes a substantial economic burden on healthcare systems (8, 9). However, the distinction between mild and severe disease is often unclear at presentation, and reliable early markers remain an unmet clinical need.

Current tools for early risk stratification of AP have well-recognized limitations. C-reactive protein (CRP), the most widely used inflammatory marker, peaks 48–72 hours after symptom onset and lacks specificity, as it can be elevated by any inflammatory or infectious stimulus (10). Established scoring systems such as Ranson criteria, Acute Physiology and Chronic Health Examination (APACHE)-II, and Bedside Index for Severity in Acute Pancreatitis (BISAP) are limited by delayed data collection, operational complexity, or parameter availability in resource-constrained settings (11). Moreover, these tools predominantly capture inflammation while overlooking insulin resistance (IR), an independent prognostic factor in AP. Given the established bidirectional interplay between inflammation and IR, a composite marker integrating both pathways may offer predictive information beyond either component alone.

Two biomarkers have attracted particular attention. CRP has been linked to AP severity and adverse outcomes (12–15). The triglyceride–glucose (TyG) index, a surrogate for IR, carries similar associations (16–18). Yet these two signals are not independent (19). Inflammation drives IR through cytokine-mediated interference with insulin signaling. In turn, IR sustains inflammation through oxidative stress and adipokine dysregulation (20). Most studies have focused on only one pathway, leaving the combined effect unexplored.

The C-reactive protein–triglyceride–glucose index (CTI) is a composite marker that combines CRP and the TyG index into a single measure (21). Originally validated in cardiovascular and metabolic disease (22), CTI has not been tested in AP. Therefore, the purpose of this study was to assess whether CTI predicts AP severity.

## Methods

### Study design and participants

This retrospective cross-sectional study included 567 patients with AP hospitalized at the First Affiliated Hospital of Sun Yat-sen University between January 2019 and January 2026. The hospital’s Ethics Committee approved the study (No (2025).268), which complied with the Declaration of Helsinki. We excluded patients under 18 years, pregnant women, those with chronic pancreatitis or pancreatic cancer, those with incomplete serum triglyceride levels, CRP, or glucose records, and readmissions within 3 months of discharge from the index admission. Such readmissions were considered part of the same clinical episode rather than a new recurrence. The selection process is shown in **Figure 1**. **Supplementary Table 1** compares baseline characteristics between patients excluded for early readmission and those included in the final cohort. Informed consent was waived because the analysis used anonymized clinical data.

**Figure 1:**
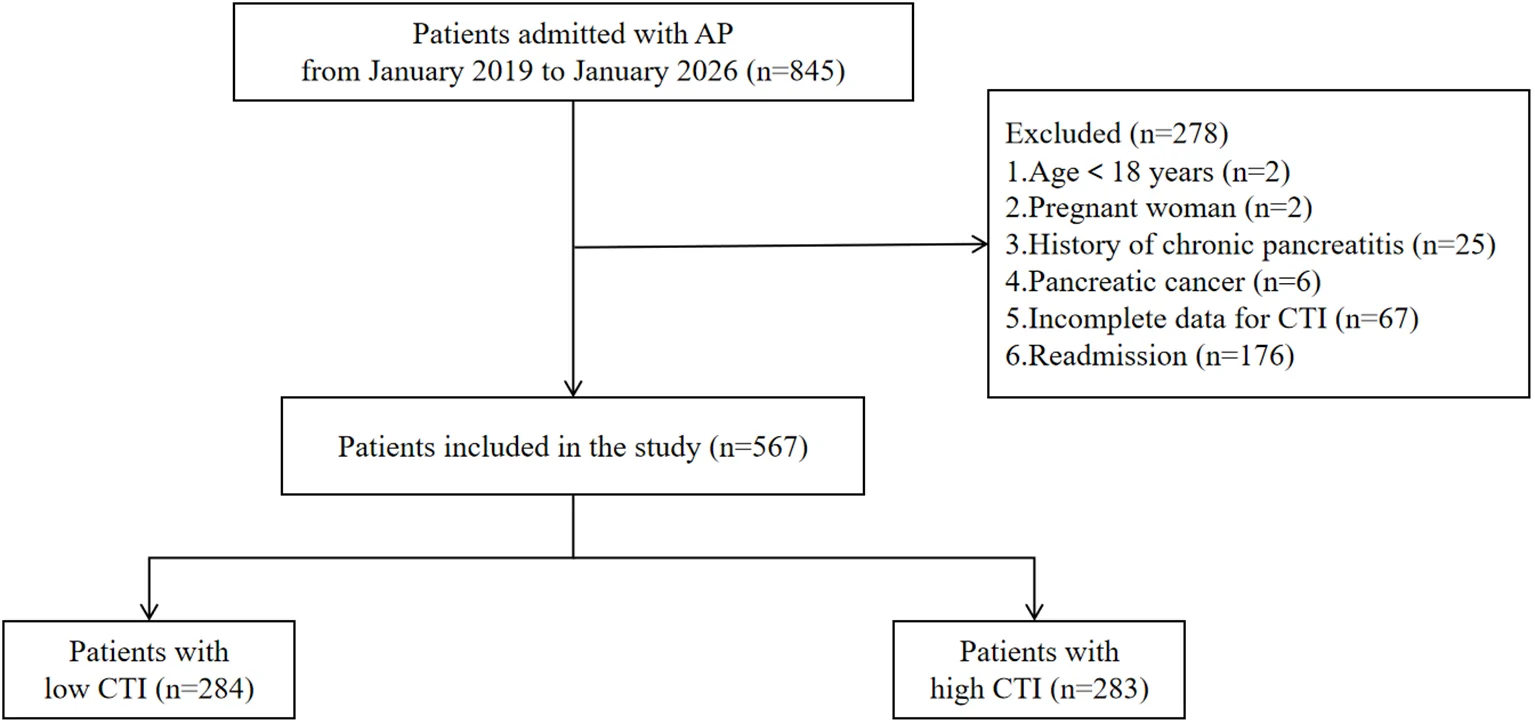
Flowchart for patient selection. AP, acute pancreatitis; CTI, C-reactive protein–triglyceride–glucose index.

### Definitions and classifications

AP diagnosis followed the 2012 revised Atlanta classification (RAC), requiring at least two of the following features: (1) characteristic abdominal pain; (2) serum amylase or lipase ≥ three times the upper reference limit; (3) imaging findings compatible with AP (23).

Etiology was classified as biliary, hypertriglyceridemic, alcoholic, other, or mixed. Biliary pancreatitis required any of: (1) imaging evidence of gallstones or bile sludge; (2) common bile duct dilation (> 8 mm if ≤ 75 years, > 10 mm if > 75 years) by ultrasound in the absence of stones or sludge; (3) alanine aminotransferase (ALT) > two times the upper limit of normal and ALT > aspartate aminotransferase (AST) (24). Hypertriglyceridemic pancreatitis was defined as AP with serum triglycerides ≥ 11.3 mmol/L at admission, after excluding other causes (25). Alcoholic pancreatitis was defined as a daily intake of approximately 150 g of alcohol over the past 10 years, with other causes ruled out (26, 27). Autoimmune, idiopathic, pancreas divisum and intraductal papillary mucinous neoplasms were grouped as other etiologies; patients with multiple coexisting causes were classified as mixed.

AP severity was assessed using both the RAC and the modified computed tomography severity index (MCTSI). Under the RAC, patients were classified as mild acute pancreatitis (MAP; no organ failure, no local or systemic complications), moderately severe acute pancreatitis (MSAP; transient organ failure < 48 hours, with local or systemic complications), or severe acute pancreatitis (SAP; organ failure persisting > 48 hours) (23, 28). We also quantified severity using the MCTSI (0–10 points), which sums scores for pancreatic inflammation (0–4), necrosis extent (0–4), and extrapancreatic complications—including pleural effusion, ascites, and peripancreatic vascular involvement (0–2) (29). MCTSI scores were stratified as mild (0–2), moderate (4–6), or severe (8–10) (29).

### CTI calculation

CTI was computed as: CTI = 0.412 × ln(CRP, mg/L) + ln[triglycerides (mg/dL) × fasting glucose (mg/dL)/2] (21).

### Data collection

We extracted demographics, smoking and alcohol status, comorbidities such as hypertension and diabetes, history of use for lipid-lowering medicine, time from symptom onset to admission, baseline laboratory parameters (complete blood count, CRP, liver and kidney function, serum lipids, pancreatic enzymes), MCTSI scores, etiology, severity of AP and length of hospital stay. CRP, fasting blood glucose, and triglyceride levels were measured from blood samples obtained within 24 hours of admission after an overnight fast of at least 8 hours. In cases where urgent samples were used, the 8-hour fasting interval was determined based on admission history documenting the time of the patient’s last oral intake. Most measurements were obtained from blood samples collected at our hospital. A minority were derived from external laboratory results documented in our medical records, provided they met the same time and fasting criteria as described above. Body mass index (BMI) was calculated as weight in kilograms divided by the square of height in meters (kg/m^2^). Laboratory technicians were blinded to clinical outcomes. Patients received standardized care according to institutional protocols.

### Statistical analysis

Continuous variables were compared using the Student’s t-test or Mann–Whitney U test, and categorical variables using the chi-square test. Missing laboratory data were imputed by multiple imputation via chained equations (20 imputed datasets, 50 iterations) under a missing-at-random assumption, with estimates pooled by Rubin’s rules. Detailed missing data patterns for continuous variables are presented in **Supplementary Table 2**. Sensitivity analyses included comparison of imputed with complete-case estimates and additional adjustment for BMI in the multivariate models to assess potential confounding of the CTI-severity association.

For baseline comparisons across the three severity grades under both the RAC and MCTSI, we used Kruskal–Wallis tests for multi-category variables, Mann–Whitney U tests for binary variables, and Spearman’s rank correlations for continuous variables. For regression analyses, we dichotomized severity into mild versus moderate-to-severe. Mild disease was defined as MAP under the RAC and as MCTSI score 0–2. Moderate-to-severe comprised all remaining cases under either grading system. Univariate logistic regression (entry threshold *P* < 0.1) screened candidate predictors (**Supplementary Table 3**). After excluding collinear variables, multivariate binary logistic regression was conducted to identify independent risk factors. To further explore the dose-response relationship between CTI and the three severity grades under both grading systems, we performed univariate ordinal logistic regression as an exploratory analysis (**Supplementary Table 4**). Receiver operating characteristic (ROC) analyses were performed for two severity endpoints—moderate-to-severe and severe—under both grading systems. Variables that were independently associated with severity in both systems were selected for ROC curve analysis, along with a combined model incorporating these markers. CRP and the TyG index, the two components of CTI, were also evaluated in the ROC analysis for comparison. Optimal cutoffs were determined using the Youden index.

Subgroup analyses were performed to further examine the association between CTI and the severity of AP across different subgroups. These subgroups were defined by sex (female and male), age (≤ 40 years, 41–60 years, and > 60 years), smoking status, drinking status, hypertension, diabetes, history of AP, and etiology (cholelithiasis, hypertriglyceridemia, alcohol, other, and mixed). All estimates were derived from multivariate logistic regression models with the same covariate adjustment as in the primary analysis. Interaction tests were conducted in the fully adjusted models to test for heterogeneity.

All tests were two-sided, with *P* < 0.05 considered significant. Analyses used SPSS software (version 27.0; Chicago, IL, USA).

## Results

### Baseline characteristics

This study included 567 AP patients (**Figure 1**). The patients were predominantly male (64.2%), with a median age of 47 (37–62) years. Comorbidities included diabetes (*n* = 122) and hypertension (*n* = 153). Patients were divided into two groups by a median split of CTI (10.56). **Table 1** provides complete group comparisons. Compared with the low-CTI group (CTI ≤ 10.56; *n* = 284), patients in the high-CTI group (CTI > 10.56; *n* = 283) were younger, predominantly male, and had longer hospital stays, along with higher BMI and higher rates of diabetes, smoking, alcohol use, and lipid-lowering therapy. Laboratory parameters, including CRP, white blood cell (WBC) count, lactate dehydrogenase (LDH), glucose, total cholesterol (TC), triglycerides, and low-density lipoprotein (LDL), were significantly elevated in the high-CTI group, whereas calcium and high-density lipoprotein (HDL) were lower (all *P* < 0.001). Biliary disease was the leading etiology, accounting for 35.8%.

**Table 1:** Comparison baseline characteristics and clinical profile between low CTI and high CTI groups.

Variables	Total (*N* = 567)	Low CTI (*n* = 284)	High CTI (*n* = 283)	*P* value
Sex				<0.001
Male (*n*, %)	364 (64.2%)	152 (53.5%)	212 (74.9%)	
Female (*n*, %)	203 (35.8%)	132 (46.5%)	71 (25.1%)	
Age (years)	47.0 (37.0–62.0)	55.0 (40.0–66.0)	43.0 (36.0–54.0)	**<0.001**
BMI (kg/m²)	24.2 (21.3–26.7)	22.7 (20.1–25.1)	25.6 (22.5–28.2)	**<0.001**
History of use for lipid-lowering medicine (*n*, %)	96 (16.9%)	35 (12.3%)	61 (21.6%)	**0.003**
History of diabetes (*n*, %)	122 (21.5%)	34 (12.0%)	88 (31.1%)	**<0.001**
History of hypertension (*n*, %)	153 (27.0%)	79 (27.8%)	74 (26.1%)	0.654
Drinking status (*n*, %)	205 (36.2%)	69 (24.3%)	136 (48.1%)	**<0.001**
Smoking status (*n*, %)	196 (34.6%)	66 (23.2%)	130 (45.9%)	**<0.001**
Time from AP attack to admission (days)	2.0 (1.0–4.0)	2.0 (1.0–3.0)	2.0 (1.0–4.0)	0.117
Etiologies				<0.001
Cholelithiasis (*n*, %)	203 (35.8%)	141 (49.6%)	62 (21.9%)	
Hypertriglyceridemia (*n*, %)	57 (10.1%)	0 (0.0%)	57 (20.1%)	
Alcohol (*n*, %)	56 (9.9%)	17 (6.0%)	39 (13.8%)	
Other (*n*, %)	201 (35.4%)	114 (40.1%)	87 (30.7%)	
Mixed (*n*, %)	50 (8.8%)	12 (4.2%)	38 (13.4%)	
Laboratory variables				
CRP (mg/L)	16.69 (4.17–105.04)	5.45 (2.02–16.51)	83.81 (18.99–197.72)	**<0.001**
WBC (×10^9/L)	11.59 (8.45–15.13)	9.93 (7.45–13.49)	12.94 (10.37–16.50)	**<0.001**
CREA (µmol/L)	65.0 (52.0–80.0)	65.0 (52.3–80.8)	64.0 (52.0–80.0)	0.832
LDH (U/L)	231.0 (189.0–334.0)	207.5 (176.0–282.5)	278.0 (207.0–385.2)	**<0.001**
Amylase (U/L)	287.0 (125.0–774.0)	453.5 (194.5–1143.9)	200.0 (79.0–525.0)	**<0.001**
Lipase (U/L)	1610.0 (493.0–3936.0)	2244.5 (910.5–4981.8)	1063.0 (379.0–2529.0)	**<0.001**
Calcium (mmol/L)	2.20 (2.10–2.31)	2.30 (2.20–2.40)	2.20 (2.00–2.30)	**<0.001**
Glucose (mmol/L)	7.20 (5.80–9.60)	6.40 (5.40–7.70)	8.50 (6.80–13.00)	**<0.001**
TC (mmol/L)	4.70 (3.72–6.34)	4.25 (3.50–4.90)	6.00 (4.20–8.50)	**<0.001**
TG (mmol/L)	1.68 (0.95–4.87)	1.01 (0.74–1.42)	4.75 (2.12–13.50)	**<0.001**
HDL (mmol/L)	0.97 (0.78–1.26)	1.06 (0.88–1.33)	0.88 (0.64–1.13)	**<0.001**
LDL (mmol/L)	2.85 (2.17–3.60)	2.58 (2.06–3.09)	3.18 (2.38–4.39)	**<0.001**
TyG index	9.24 (8.53–10.49)	8.54 (8.22–8.97)	10.49 (9.52–11.63)	**<0.001**
CTI	10.56 (9.41–11.92)	9.42 (8.82–9.98)	11.92 (11.10–13.19)	**<0.001**
Severity of AP				<0.001
MAP (*n*, %)	299 (52.7%)	185 (65.1%)	114 (40.3%)	
MSAP (*n*, %)	222 (39.2%)	90 (31.7%)	132 (46.6%)	
SAP (*n*, %)	46 (8.1%)	9 (3.2%)	37 (13.1%)	
MCTSI				<0.001
Mild (*n*, %)	254 (44.8%)	169 (59.5%)	85 (30.0%)	
Moderate (*n*, %)	263 (46.4%)	100 (35.2%)	163 (57.6%)	
Severe (*n*, %)	31 (5.5%)	4 (1.4%)	27 (9.5%)	
Not assessable (*n*, %)	19 (3.4%)	11 (3.9%)	8 (2.8%)	
Length of stay (days)	7.0 (5.0–11.0)	6.0 (4.0–9.0)	8.0 (5.0–14.0)	**<0.001**

Values are presented as means ± standard deviation, median (interquartile range) or *n* (%). Mild (MCTSI score 0 and 2), moderate (MCTSI score 4 and 6), severe (MCTSI score 8 and 10). *P* < 0.05 was bolded.

CTI, C-reactive protein–triglyceride–glucose index; BMI, body mass index; AP, acute pancreatitis; CRP, C-reactive protein; WBC, white blood cell; CREA, creatinine; LDH, lactate dehydrogenase; TC, total cholesterol; TG, triglyceride; HDL, high-density lipoprotein; LDL, low-density lipoprotein; TyG index, triglyceride–glucose index; MAP, mild acute pancreatitis; MSAP, moderately severe acute pancreatitis; SAP, severe acute pancreatitis; MCTSI, the modified computed tomography severity index.

### CTI and AP severity

Under the RAC, the proportion of patients with MSAP and SAP was 31.7% and 3.2% in the low-CTI group, compared with 46.6% and 37.0% in the high-CTI group (*P* < 0.001; **Table 1**). The association between CTI and severity was consistent under both the RAC and MCTSI grading systems (both *P* < 0.001; **Table 1**).

Spearman’s rank correlation confirmed a positive association between CTI and severity under both grading systems (both *P* < 0.001; **Table 2**). In exploratory univariate ordinal logistic regression, CTI showed a significant dose-response association with increasing severity grades under both the RAC (odds ratio [OR] 1.282, 95% confidence interval [CI] 1.172–1.402, *P* < 0.001) and MCTSI (OR 1.476, 95% CI 1.338–1.628, *P* < 0.001) classifications (**Supplementary Table 4**). In multivariate regression, CTI remained an independent predictor of severity under the RAC (OR 1.180, 95% 95% CI 1.027–1.357, *P* = 0.019; **Table 3**) and MCTSI (OR 1.436, 95% CI 1.237–1.667, *P* < 0.001; **Table 3**).

**Table 2:** Association of variables with severity of AP using different statistical methods.

Variable	Severity (RAC)		Severity (MCTSI)	*P* value
	Statistic	*P* value	Statistic	
Categorical variables				
Etiology	H = 16.163, df = 4	**0.003**	H = 25.035, df = 4	**<0.001**
Sex (male/female)	U = 32586.5, Z = –2.618	**0.009**	U = 30359.5, Z = –2.756	**0.006**
History of use for lipid-lowering medicine (yes/no)	U = 23186.0, Z = 0.444	0.657	U = 22104.5, Z = 0.766	0.444
Smoking status (yes/no)	U = 41042.0, Z = 2.836	**0.005**	U = 39634.5, Z = 3.588	**<0.001**
Drinking status (yes/no)	U = 42414.0, Z = 3.181	**0.001**	U = 40319.5, Z = 3.531	**<0.001**
History of diabetes (yes/no)	U = 28295.5, Z = 0.806	0.420	U = 27112.5, Z = 1.287	0.198
History of hypertension (yes/no)	U = 31548.0, Z = –0.080	0.936	U = 27207.5, Z = –1.210	0.226
Continuous variables				
Age	ρ = –0.091	**0.029**	ρ = –0.107	**0.013**
BMI	ρ = 0.000	0.994	ρ = 0.053	0.211
Hospital stay	ρ = 0.363	**<0.001**	ρ = 0.349	**<0.001**
CRP	ρ = 0.262	**<0.001**	ρ = 0.340	**<0.001**
WBC	ρ = 0.146	**<0.001**	ρ = 0.220	**<0.001**
CREA	ρ = 0.020	0.636	ρ = –0.007	0.870
LDH	ρ = 0.206	**<0.001**	ρ = 0.238	**<0.001**
Amylase	ρ = –0.082	0.0504	ρ = –0.127	**0.003**
Lipase	ρ = –0.110	**0.009**	ρ = –0.152	**<0.001**
Calcium	ρ = –0.242	**<0.001**	ρ = –0.255	**<0.001**
Glucose	ρ = 0.079	0.061	ρ = 0.152	**<0.001**
TC	ρ = –0.003	0.951	ρ = 0.065	0.127
TG	ρ = 0.151	**<0.001**	ρ = 0.236	**<0.001**
HDL	ρ = –0.189	**<0.001**	ρ = –0.205	**<0.001**
LDL	ρ = 0.016	0.702	ρ = 0.077	0.072
TyG index	ρ = 0.155	**<0.001**	ρ = 0.247	**<0.001**
CTI	ρ = 0.240	**<0.001**	ρ = 0.337	**<0.001**

Categorical variables were analyzed using Kruskal-Wallis H test (etiology) or Mann-Whitney U test (binary variables); continuous variables were analyzed using Spearman’s rank correlation. Etiology categories comprise biliary, hypertriglyceridemic, alcoholic, other, and mixed etiologies. *P* < 0.05 was bolded.

AP, acute pancreatitis; RAC, revised Atlanta criteria; MCTSI, modified computed tomography severity index; BMI, body mass index; CRP, C-reactive protein; WBC, white blood cell; CREA, creatinine; LDH, lactate dehydrogenase; TC, total cholesterol; TG, triglyceride; HDL, high-density lipoprotein; LDL, low-density lipoprotein; TyG index, triglyceride–glucose index; CTI, C-reactive protein–triglyceride–glucose index.

**Table 3:** Multivariable logistic regression analysis for severity of AP.

Variable	Severity (RAC)		Severity (MCTSI)	
	OR (95% CI)	*P* value	OR (95% CI)	*P* value
Sex (male/female)	1.222 (0.788–1.895)	0.371	0.949 (0.600–1.501)	0.824
Age	0.995 (0.993–1.007)	0.369	0.999 (0.987–1.011)	0.848
Smoking status (yes/no)	1.102 (0.681–1.783)	0.693	0.794 (0.477–1.322)	0.375
Drinking status (yes/no)	1.150 (0.681–1.941)	0.600	0.944 (0.543–1.641)	0.839
WBC	1.025 (0.988–1.064)	0.18	1.050 (1.009–1.092)	**0.015**
LDH	1.001 (1.000–1.002)	**0.009**	1.001 (1.000–1.002)	**0.048**
Calcium	0.669 (0.310–1.445)	0.305	0.729 (0.328–1.620)	0.436
CTI	1.180 (1.027–1.357)	**0.019**	1.436 (1.237–1.667)	**<0.001**
Etiology (ref: Cholelithiasis)		0.284		0.135
Hypertriglyceridemia	0.927 (0.410–2.094)	0.856	0.485 (0.203–1.157)	0.102
Alcohol	1.637 (0.773–3.468)	0.198	1.617 (0.723–3.617)	0.243
Other	0.990 (0.646–1.517)	0.963	0.829 (0.531–1.293)	0.408
Mixed	0.639 (0.289–1.412)	0.268	0.656 (0.283–1.518)	0.324

OR, odds ratio; CI, confidence interval. *P* < 0.05 was bolded.

AP, acute pancreatitis; RAC, revised Atlanta criteria; MCTSI, modified computed tomography severity index; WBC, white blood cell; LDH, lactate dehydrogenase; CTI, C-reactive protein–triglyceride–glucose index.

The discriminative performance of CTI for AP severity was evaluated using ROC curve analysis (**Figure 2**). For RAC-defined SAP, CTI yielded an area under the ROC curve (AUC) of 0.680 (95% CI 0.606–0.755), with a cutoff of 10.90 (sensitivity 89.1%, specificity 52.8%). For MCTSI ≥ 8, the AUC was 0.763 (95% CI 0.681–0.845) at the same cutoff (sensitivity 87.1%, specificity 59.8%), outperforming CRP (AUC 0.746). For moderate-to-severe disease, the AUCs were 0.628 (RAC-defined MSAP/SAP) and 0.681 (MCTSI ≥ 4). In all comparisons, CTI outperformed the TyG index.

**Figure 2:**
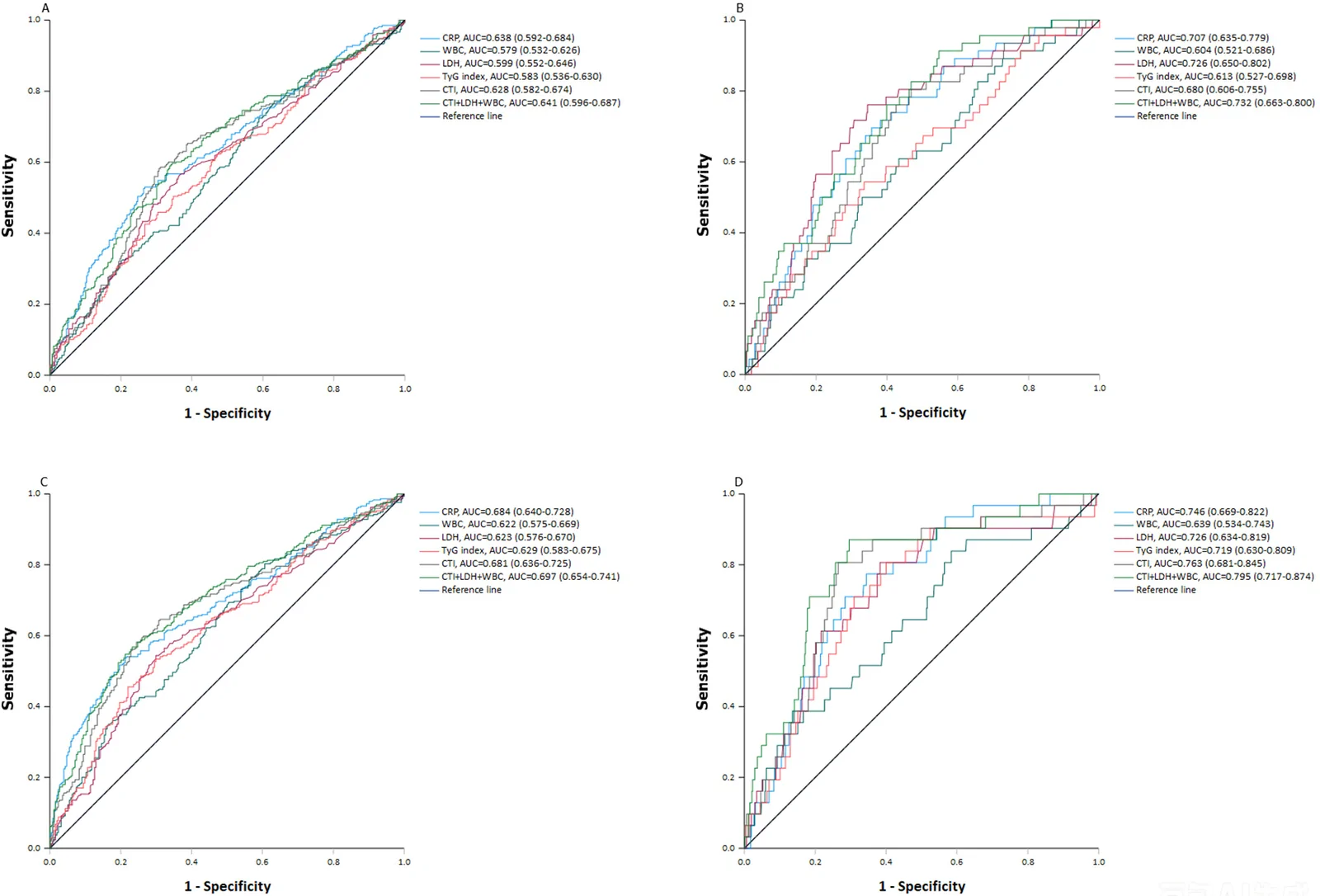
ROC curves of individual biomarkers and combined model for predicting the severity of AP. **(A)** For predicting MSAP and SAP according to the revised Atlanta classification; **(B)** For predicting SAP according to the revised Atlanta classification; **(C)** For predicting moderate and severe AP (MCTSI score ≥ 4) according to the MCTSI classification; **(D)** For predicting SAP (MCTSI score ≥ 8) according to the MCTSI classification. The dashed diagonal line represents the reference (AUC = 0.5). ROC, receiver operating characteristic; AP, acute pancreatitis; CRP, C-reactive protein; WBC, white blood cell; LDH, lactate dehydrogenase; TyG index, triglyceride–glucose index; CTI, C-reactive protein–triglyceride–glucose index; MCTSI, modified computed tomography severity index. Combined model, a logistic regression-based combination of CTI, LDH, and WBC; MSAP, moderately severe acute pancreatitis; SAP, severe acute pancreatitis; MCTSI, modified computed tomography severity index; AUC, the area under the receiver operating characteristic curve.

The combined model (CTI + WBC + LDH) achieved the highest AUCs across all endpoints. Under the MCTSI, the AUCs were 0.697 for moderate-to-severe disease (MCTSI ≥ 4) and 0.795 for severe disease (MCTSI ≥ 8). Under the RAC, the AUCs were 0.641 for moderate-to-severe disease (MSAP/SAP) and 0.732 for SAP.

### Subgroup analyses and interaction tests

Estimates for individual subgroups are presented in **Table 4**. The association between CTI and AP severity remained consistent across most strata, including sex, BMI, smoking status, hypertension, diabetes and etiology (all *P* for interaction > 0.05). Under the RAC, drinking status modified the association (*P* for interaction = 0.014): CTI was associated with severity in non-drinkers (OR 1.407, *P* < 0.001), whereas no significant association was observed in drinkers (OR 0.908, *P* = 0.419). Under the MCTSI, age stratified the association (*P* for interaction = 0.045): CTI was associated with severity in patients aged 41–60 years (OR 1.570, *P* < 0.001) and > 60 years (OR 1.714, *P* = 0.005), but not in those ≤ 40 years (OR 1.172, *P* = 0.219).

**Table 4:** Subgroup analysis of the association between CTI and severity of AP.

Subgroup	Severity (RAC)		*P* for interaction	Severity (MCTSI)		*P* for interaction
	OR (95% CI)	*P* value		OR (95% CI)	*P* value	
Sex			0.072			0.575
Female	1.541 (1.168–2.031)	**0.002**		1.757 (1.320–2.340)	**<0.001**	
Male	1.025 (0.866–1.213)	0.778		1.288 (1.071–1.549)	**0.007**	
Age group			0.116			0.045
≤ 40 years	1.096 (0.861–1.396)	0.458		1.172 (0.910–1.510)	0.219	
41–60 years	1.153 (0.927–1.434)	0.197		1.570 (1.223–2.014)	**<0.001**	
> 60 years	1.337 (0.950–1.882)	0.095		1.714 (1.179–2.493)	**0.005**	
BMI group						
< 18.5	3.317 (1.292–8.512)	**0.013**	0.581	2.627 (1.170–5.897)	**0.019**	0.786
18.5–23.9	1.208 (0.973–1.500)	0.087		1.523 (1.197–1.937)	**0.001**	
24.0-27.9	1.197 (0.906–1.582)	0.205		1.346 (1.006–1.800)	**0.045**	
≥ 28	1.141 (0.747–1.743)	0.541		2.026 (1.200–3.419)	**0.008**	
Smoking status			0.086			0.71
No	1.405(1.166–1.693)	**<0.001**		1.537 (1.263–1.872)	**<0.001**	
Yes	0.897 (0.713–1.128)	0.350		1.240 (0.963–1.596)	0.096	
Drinking status			0.014			0.644
No	1.407 (1.166–1.697)	**<0.001**		1.552 (1.276–1.889)	**<0.001**	
Yes	0.908 (0.717–1.149)	0.419		1.247 (0.959–1.621)	0.100	
Hypertension			0.349			0.208
No	1.162 (0.989–1.366)	0.068		1.446 (1.214–1.723)	**<0.001**	
Yes	1.311 (0.972–1.769)	0.076		1.558 (1.117–2.173)	**0.009**	
Diabetes			0.587			0.677
No	1.237 (1.053–1.453)	**0.009**		1.502 (1.261–1.790)	**<0.001**	
Yes	1.015 (0.697–1.479)	0.938		1.424 (0.947–2.142)	0.089	
Etiology			0.795			0.794
Cholelithiasis	1.182 (0.890–1.570)	0.249		1.415 (1.053–1.901)	**0.021**	
Hypertriglyceridemia	1.025 (0.575–1.827)	0.933		1.512 (0.809–2.827)	0.195	
Alcohol	0.808 (0.443–1.475)	0.487		1.930 (0.916–4.069)	0.084	
Other	1.285 (1.036–1.594)	**0.023**		1.399 (1.117–1.753)	**0.004**	
Mixed	0.984 (0.640–1.514)	0.94		1.169 (0.682–2.003)	0.571	

Data were adjusted for sex, age, smoking, drinking, WBC, LDH, calcium, and etiology in the multivariate logistic regression models, except for the stratification variable itself in each subgroup analysis. OR, odds ratio; CI, confidence interval. *P* < 0.05 was bolded.

CTI, C-reactive protein–triglyceride–glucose index; AP, acute pancreatitis; RAC, revised Atlanta criteria; MCTSI, modified computed tomography severity index; BMI, body mass index; WBC, white blood cell; LDH, lactate dehydrogenase.

### Other predictors of AP severity

Univariate analyses for additional variables (**Table 2**) were generally consistent across both grading systems. Etiology, male sex, smoking, drinking, hospital stay, CRP, WBC, LDH, triglycerides, and the TyG index all positively associated with severity (all *P* < 0.05); age, lipase, calcium, and HDL negatively associated. Glucose and amylase also showed significant associations only under the MCTSI (*P* < 0.05). BMI, hypertension, diabetes, creatinine, TC, LDL and history of use for lipid-lowering medicine were not significant (*P* > 0.05).

In multivariate models (**Table 3**), independent risk factors varied by grading system. Under the RAC, prior AP history (OR 1.76, 95% CI 1.15–2.70, *P* = 0.010) and LDH (OR 1.001, 95% CI 1.000–1.002, *P* = 0.005) were independent risk factors for severity. Under the MCTSI, WBC (OR 1.05, 95% CI 1.01–1.09, *P* = 0.015) and LDH (OR 1.001, 95% CI 1.000–1.002, *P* = 0.048) remained independent predictors. Notably, LDH showed consistent predictive performance across both grading systems: ROC analysis (**Figure 2**) yielded an AUC of 0.726 for predicting SAP under the RAC, with an optimal cutoff of 277 U/L (sensitivity 87.1%, specificity 64.2%). For MCTSI ≥ 8, the AUC was also 0.726, with a cutoff of 292 U/L (sensitivity 84.8%, specificity 61.6%).

### Sensitivity analysis

To assess the robustness of our findings, we compared the results from multiple imputation with those from complete-case results. The direction, magnitude, and statistical significance of the associations between CTI and AP severity were consistent across both approaches, supporting the validity of our primary findings. Detailed results of the sensitivity analyses are presented in **Supplementary Table 5**, **6**. We further performed a sensitivity analysis with additional adjustment for BMI, in which CTI remained an independent predictor of AP severity under both the RAC (OR 1.25, 95% CI 1.08–1.45, *P* = 0.003) and MCTSI (OR 1.51, 95% CI 1.29–1.77, *P* < 0.001) classifications (**Supplementary Table 7**).

## Discussion

In this retrospective study, we found that CTI—a novel composite biomarker integrating CRP and the TyG index—independently predicted AP severity. This association was consistent across both the RAC and MCTSI grading systems, with stronger discriminative ability for MCTSI-defined SAP (AUC 0.763). To the best of our knowledge, this is the first study to report an association between CTI and the severity of AP.

CTI provided incremental predictive value beyond its individual components. It consistently outperformed the TyG index across all severity endpoints, and showed a modest advantage over CRP in MCTSI-defined severe AP. This advantage likely reflects the synergistic interplay between inflammation and IR—two interconnected processes that neither marker captures in isolation. By integrating both signals, CTI captures a broader spectrum of metabolic-inflammatory dysregulation, consistent with evidence from other disease contexts (30–32).

The association between CTI and AP may be explained by two interconnected processes: inflammation and IR. CRP amplifies inflammation through complement C1q activation and fragment crystallizable receptor engagement, triggering pro-inflammatory cytokine release (33). Elevated CRP levels at a cutoff of 150 mg/L have been reported to yield a sensitivity of 53% and specificity of 82% for predicting the severity of AP (34). IR, meanwhile, sets the metabolic stage for tissue injury. It promotes hypertriglyceridemia, which exacerbates hyperglycemia and, in severe cases, can precipitate ketoacidosis (35). Hyperglycemia drives ROS production, directly damaging mitochondria and activating inflammatory cascades (36). It also impairs microvascular function and precipitates pancreatic ischemia (36). Hypertriglyceridemia elevates circulating free fatty acids (FFAs), which inhibit acinar cell mitochondrial complexes, induce calcium overload, promote cytokine release, cause tissue damage and pancreatic duct dysfunction (37). At high concentrations, FFAs can precipitate multi-organ failure (37). The TyG index, a well-validated surrogate for IR, reflects this metabolic burden and has shown superior predictive value over conventional lipid parameters for SAP (38–41).

By integrating CRP and the TyG index, CTI provides a composite assessment of both inflammatory and IR status. These two processes may feed into a vicious cycle. Tumor necrosis factor-alpha (TNF-α), interleukin-6 (IL-6), and leptin drive IR (42–44), while oxidative stress, inflammatory cytokine release and adipokine dysregulation perpetuate inflammation (20). IR initially arises from dysfunction in white adipose tissue and circulating metabolic disturbances (45). However, when energy imbalance becomes chronic, inflammatory pathways are activated (45). Obesity-associated low-grade systemic inflammation, particularly in adipose tissue and liver, drives IR and glycemic impairment (46). Dysregulated immune responses resulting from gut barrier disruption further compound these metabolic disturbances (46). Recent evidence indicates that adipose tissue releases multiple excess metabolic mediators, including adipokines, cytokines, chemokines, excess lipids, and toxic metabolites, all of which further impair peripheral insulin responsiveness (16). Elevated TyG index correlates with increased CRP, IL-6, chemokines, and endotoxins, along with oxidative stress markers such as oxidized LDL and nitrotyrosine (47). Toll-like receptors (TLRs) 4 and 2 on monocytes, as well as their downstream signaling pathways, are also activated (47). Collectively, these observations underscore the close relationship between IR and a pro-inflammatory, pro-oxidative state.

A single biomarker cannot fully capture the complexity of this interplay. Joint assessment of multiple indices can uncover interactions that individual markers obscure. Given the substantial heterogeneity in biomarker levels between individuals, the TyG index and CRP provide complementary rather than redundant information (48). Their integration improves risk stratification by encompassing a broader spectrum of metabolic-inflammatory dysregulation. CTI is a simple, readily available index that has been used to characterize the inflammatory-metabolic burden across several chronic diseases (22, 49). Our finding that CTI independently predicts AP severity across both grading systems is biologically plausible and extends the utility of this index to AP.

The combined model incorporating CTI, WBC, and LDH achieved the highest AUCs across all severity endpoints. This suggests that integrating multiple biomarkers may offer a more comprehensive reflection of the underlying pathophysiological processes in AP. WBC and LDH were independently associated with AP severity in our study, consistent with prior studies linking these markers to SAP and mortality (50–52). LDH also correlated with infected pancreatic necrosis (OR 1.001, 95% CI 1.000–1.003, *P* = 0.028) (53). However, the improvement over CTI alone was modest (AUC gains 0.016–0.032). This may be attributed to the relatively small number of severe cases in our study, which limited our ability to detect a more substantial difference. Despite its modest incremental gain, CTI adds clinically useful information to existing risk stratification tools and clinical assessment. Future studies with larger sample sizes are needed to determine whether other biomarkers could provide greater incremental value when combined with CTI.

Sensitivity analysis with additional adjustment for BMI confirmed that the association between CTI and AP severity was not confounded by overall adiposity. CTI remained a significant independent predictor after BMI adjustment. Notably, BMI itself showed an inverse association with severity in the adjusted models. This finding is consistent with the “obesity paradox” described in acute pancreatitis. where higher BMI may paradoxically be associated with better outcomes (54). However, this paradox may reflect the limitations of BMI as a crude measure of adiposity, as it cannot distinguish between visceral and subcutaneous adipose tissue, nor between fat and muscle (55). In acute pancreatitis, visceral adipose tissue (VAT) has been implicated in disease severity through its pro-inflammatory and lipotoxic effects, and its accumulation has been associated with heightened systemic inflammation in hyperinflammatory conditions (55). Although our study did not directly measure VAT, the observation that BMI did not confound the CTI–severity association suggests that CTI captures a broader inflammatory–metabolic signal beyond overall obesity. Future studies incorporating direct measurements of VAT may provide more precise insights into the relationship between adiposity and AP severity.

We further examined whether the association between CTI and AP severity varied across clinically relevant subgroups. Under the RAC, CTI was associated with severity in non-drinkers but not in drinkers. This discrepancy likely reflects a reflection of the influence of alcohol on AP. Overuse of alcohol is a risk factor, accounting for approximately 21% of AP cases (56). Chronic exposure increases pancreatic vulnerability and, in combination with other triggers, exacerbates disease severity (57). The direct toxic effects of alcohol metabolites on acinar cells may override the inflammatory–metabolic signals captured by CTI, attenuating its predictive value in drinkers. Under the MCTSI, CTI predicted severity in older patients, a pattern consistent with the known effects of aging on AP outcomes. Older individuals typically present with greater metabolic and inflammatory dysregulation (58), which may amplify the predictive signal of CTI. No other interactions reached significance, indicating that CTI performance remained consistent across strata of sex, smoking, hypertension, diabetes, history of AP, and etiology.

Several limitations should be acknowledged. The retrospective design precludes causal inference, and subgroup analyses should be interpreted with caution given the limited sample sizes in certain strata. Although missing data were handled by multiple imputation, we could not compare excluded patients with the included cohort, and residual bias may remain if missingness was not completely at random. CTI was measured only at admission, without serial monitoring during hospitalization. We are unable to comment on its dynamic trajectory during the acute course. Waist circumference was not available in this dataset, and some laboratory measurements, particularly glucose and lipid levels, may have been influenced by pre-sampling intravenous fluid administration. The small number of severe AP cases precluded more granular ordinal or multinomial regression analyses. The absence of external validation and the single-center design further limit the generalizability of our findings to broader populations and practice settings. Residual confounding from unmeasured variables cannot be excluded. Prospective, multicenter studies with longitudinal sampling are needed to further validate and extend these observations.

Despite these limitations, CTI may offer practical utility. It can be calculated from routinely available admission laboratory data, without requiring specialized assays or additional cost. Should the observed improvements in risk stratification be validated in future studies, CTI could serve as a useful adjunct to clinical assessment.

## Conclusion

CTI independently predicts AP severity with moderate discriminative accuracy. Its routine availability and low cost support its potential as a practical adjunct for early risk stratification. Adding WBC and LDH to CTI yielded the highest AUCs, though the incremental improvement over CTI alone was limited. Prospective studies with larger and more diverse cohorts are warranted to evaluate its integration into clinical decision-making.

## Data Availability

The raw data supporting the conclusions of this article will be made available by the authors, without undue reservation.
