# Supplementary material for: C-reactive protein–triglyceride–glucose index predicts acute pancreatitis severity: a retrospective cross-sectional study
Source: Front Endocrinol (Lausanne). 2026 Sep 21;17:1964371. doi: 10.3389/fendo.2026.1964371 (PMC13635158; doi:10.3389/fendo.2026.1964371)
Supplement: Supplementary file 1 [file Table1.docx]

Supplementary Table 1. Baseline characteristics of included patients and patients excluded for readmission for the same episode

| **Variables** | **Included group (*n* = 567)** | **Readmission-excluded group (*n* = 98)** | ***P* value** |
| --- | --- | --- | --- |
| Sex |  |  | **0.047** |
| Male (n, %) | 364 (64.2%) | 73（74.5%） |  |
| Female (n, %) | 203 (35.8%) | 25（25.5%） |  |
| Age (years) | 47.0 (37.0–62.0) | 42.5 (35.0–53.0) | **0.005** |
| BMI (kg/m²) | 24.2 (21.3–26.7) | 24.6 (21.9–26.9) | 0.448 |
| History of use for lipid-lowering medicine (n, %) | 96 (16.9%) | 24 (24.5%) | 0.072 |
| History of diabetes (n, %) | 122 (21.5%) | 44 (44.9%) | **0.049** |
| History of hypertension (n, %) | 153 (27.0%) | 47 (48.0%) | **0.026** |
| Drinking status (n, %) | 205 (36.2%) | 22 (22.4%) | 0.836 |
| Smoking status (n, %) | 196 (34.6%) | 25 (25.5%) | 0.761 |
| Time from AP attack to admission (days) | 2.0 (1.0–4.0) | 2.0 (1.0–3.0) | 0.287 |
| Etiologies |  |  | 0.076 |
| Cholelithiasis (n, %) | 203 (35.8%) | 25 (25.5%) |  |
| Hypertriglyceridemia (n, %) | 57 (10.1%) | 14 (14.3%) |  |
| Alcohol (n, %) | 56 (9.9%) | 14 (14.3%) |  |
| Other (n, %) | 201 (35.4%) | 31 (31.6%) |  |
| Mixed (n, %) | 50 (8.8%) | 14 (14.3%) |  |
| Laboratory variables |  |  |  |
| CRP (mg/L) | 16.69 (4.17–105.04) | 17.71 (7.67–144.42) | 0.233 |
| WBC (×10^9/L) | 11.59 (8.45–15.13) | 11.64 (9.85–16.96) | 0.085 |
| CREA (µmol/L) | 65.0 (52.0–80.0) | 66.0 (55.0–77.0) | 0.913 |
| LDH (U/L) | 231.0 (189.0–334.0) | 231.5 (179.5–334.5) | 0.656 |
| Amylase (U/L) | 287.0 (125.0–774.0) | 315.5 (117.0–846.0) | 0.943 |
| Lipase (U/L) | 1610.0 (493.0–3936.0) | 1873.0 (452.0–4346.0) | 0.775 |
| Calcium (mmol/L) | 2.20 (2.10–2.31) | 2.20 (2.10–2.30) | 0.319 |
| Glucose (mmol/L) | 7.20 (5.80–9.60) | 7.30 (5.80–10.40) | 0.688 |
| TC (mmol/L) | 4.70 (3.72–6.34) | 4.90 (4.00–7.40) | 0.09 |
| TG (mmol/L) | 1.68 (0.95–4.87) | 2.57 (1.01–9.63) | 0.05 |
| HDL (mmol/L) | 0.97 (0.78–1.26) | 0.97 (0.72–1.19) | 0.477 |
| LDL (mmol/L) | 2.85 (2.17–3.60) | 2.98 (2.32–3.86) | 0.271 |
| TyG index | 9.24 (8.53–10.49) | 9.66 (8.69–11.15) | **0.04** |
| CTI | 10.56 (9.41–11.92) | 11.02 (9.61–12.83) | **0.045** |
| Severity of AP |  |  | 0.57 |
| MAP (n, %) | 299 (52.7%) | 46 (46.9%) |  |
| MSAP (n, %) | 222 (39.2%) | 43 (43.9%) |  |
| SAP (n, %) | 46 (8.1%) | 9 (9.2%) |  |
| MCTSI |  |  | **0.035** |
| Mild (n, %) | 254 (44.8%) | 42 (42.9%) |  |
| Moderate (n, %) | 263 (46.4%) | 41 (41.8%) |  |
| Severe (n, %) | 31 (5.5%) | 13 (13.3%) |  |
| Not assessable (n, %) | 19 (3.4%) | 2 (2.0%) |  |
| Length of stay (days) | 7.0 (5.0–11.0) | 7.0 (5.0–10.3) |  |

Notes: Values are presented as means ± standard deviation, median (interquartile range) or *n* (%). Mild (MCTSI score 0 and 2), moderate (MCTSI score 4 and 6), severe (MCTSI score 8 and 10). *P* < 0.05 was bolded. The readmission-excluded group comprised 176 patients who were excluded for readmission for the same episode; after removing duplicate records for patients with multiple readmissions within the 3-month window, 98 unique patients were identified, and their data at the index admission are presented.

Abbreviations: BMI, body mass index; AP, acute pancreatitis; CRP, C-reactive protein; WBC, white blood cell; CREA, creatinine; LDH, lactate dehydrogenase; TC, total cholesterol; TG, triglyceride; HDL, high-density lipoprotein; LDL, low-density lipoprotein; TyG index, triglyceride–glucose index; CTI, C-reactive protein–triglyceride–glucose index; MAP, mild acute pancreatitis; MSAP, moderately severe acute pancreatitis; SAP, severe acute pancreatitis; MCTSI, the modified computed tomography severity index.

Supplementary Table 2. Missing data pattern of continuous variables

| **Variables** | ***N*** | **Mean ± SD** | **Missing *n* (%)** |
| --- | --- | --- | --- |
| Age (years) | 567 | 49.81 ± 16.41 | 0 (0.0) |
| Height (cm) | 505 | 165.74 ± 8.09 | 62 (10.9%) |
| Weight (kg) | 505 | 66.78 ± 14.73 | 62 (10.9%) |
| Time from AP attack to admission (days) | 567 | 3.76 ± 5.39 | 0 (0.0) |
| Length of stay (days) | 567 | 9.67 ± 9.94 | 0 (0.0) |
| CRP (mg/L) | 567 | 69.46 ± 97.47 | 0 (0.0) |
| WBC (×10^9/L) | 567 | 12.19 ± 4.99 | 0 (0.0) |
| CREA (µmol/L) | 567 | 85.28 ± 101.86 | 0 (0.0) |
| LDH (U/L) | 561 | 323.99 ± 276.69 | 6 (1.1) |
| Amylase (U/L) | 567 | 689.39 ± 955.21 | 0 (0.0) |
| Lipase (U/L) | 567 | 3018.21 ± 4475.67 | 0 (0.0) |
| Calcium (mmol/L) | 562 | 2.22 ± 0.31 | 5 (0.9) |
| Glucose (mmol/L) | 567 | 8.53 ± 4.29 | 0 (0.0) |
| TC (mmol/L) | 564 | 5.79 ± 3.89 | 3 (0.5) |
| TG (mmol/L) | 567 | 6.89 ± 17.11 | 0 (0.0) |
| HDL (mmol/L) | 543 | 1.06 ± 0.63 | 24 (4.2) |
| LDL (mmol/L) | 548 | 3.02 ± 1.36 | 19 (3.4) |
| TyG index | 567 | 9.60 ± 1.45 | 0 (0.0) |
| CTI | 567 | 10.80 ± 1.85 | 0 (0.0) |

Notes: *N* indicates the number of non‑missing cases for each variable. Continuous variables are presented as mean ± standard deviation (SD). Missing *n* (%) refers to the number (and percentage) of missing observations.

Abbreviations: AP, acute pancreatitis; CRP, C-reactive protein; WBC, white blood cell; CREA, creatinine; LDH, lactate dehydrogenase; TC, total cholesterol; TG, triglyceride; HDL, high-density lipoprotein; LDL, low-density lipoprotein; TyG index, triglyceride-glucose index; CTI, C-reactive protein–triglyceride–glucose index.

Supplementary Table 3. Univariate logistic regression analysis for severity (RAC) and severity (MCTSI)

| **Variable** | **Severity (RAC）** |  | **Severity (MCTSI)** |  |
| --- | --- | --- | --- | --- |
|  | **OR (95% CI)** | ***P* value** | **OR (95% CI)** | ***P* value** |
| Sex (male/female) | 1.59 (1.12–2.25) | **0.009** | 1.50 (1.06–2.13) | **0.023** |
| Age | 0.99 (0.98–1.00) | **0.015** | 0.99 (0.98–1.00) | 0.055 |
| BMI | 0.995 (0.956 – 1.034) | 0.791 | 1.029 (0.987 – 1.072) | 0.176 |
| History of use for lipid-lowering medicine (yes/no) | 1.14 (0.74–1.77) | 0.556 | 1.12 (0.71–1.75) | 0.631 |
| Smoking status (yes/no) | 1.62 (1.14–2.29) | **0.007** | 1.75 (1.22–2.51) | **0.002** |
| Drinking status (yes/no) | 1.69 (1.20–2.39) | **0.003** | 1.74 (1.22–2.48) | **0.002** |
| Diabetes (yes/no) | 1.15 (0.77–1.72) | 0.495 | 1.40 (0.93–2.12) | 0.11 |
| Hypertension (yes/no) | 0.92 (0.63–1.34) | 0.661 | 0.77 (0.53–1.14) | 0.19 |
| CRP | 1.005 (1.003–1.007) | **<0.001** | 1.009 (1.007–1.012) | **<0.001** |
| WBC | 1.05 (1.02–1.09) | **0.004** | 1.09 (1.05–1.13) | **<0.001** |
| CREA | 1.001 (0.999–1.003) | 0.238 | 1.00 (0.99–1.00) | 0.606 |
| LDH | 1.001 (1.001–1.002) | **0.001** | 1.001 (1.001–1.002) | **0.001** |
| Amylase | 1.00 (1.00–1.00) | 0.909 | 1.00 (1.00–1.00) | 0.568 |
| Lipase | 1.00 (1.00–1.00) | 0.56 | 1.00 (1.00–1.00) | 0.24 |
| Calcium | 0.34 (0.16–0.72) | **0.005** | 0.28 (0.13–0.62) | **0.002** |
| Glucose | 1.03 (0.99–1.07) | 0.106 | 1.08 (1.04–1.13) | **0.001** |
| TC | 1.05 (1.01–1.10) | **0.023** | 1.10 (1.04–1.16) | **<0.001** |
| TG | 1.02 (1.01–1.03) | **0.006** | 1.03 (1.01–1.05) | **0.002** |
| HDL | 0.86 (0.66–1.11) | 0.251 | 0.90 (0.70–1.15) | 0.399 |
| LDL | 1.20 (1.06–1.35) | **0.003** | 1.28 (1.12–1.46) | **<0.001** |
| TyG index | 1.23 (1.09–1.38) | **0.001** | 1.40 (1.23–1.59) | **<0.001** |
| CTI | 1.27 (1.16–1.40) | **<0.001** | 1.46 (1.31–1.62) | **<0.001** |
| High CTI (yes/no) | 2.77 (1.97–3.89) | **<0.001** | 3.64 (2.55–5.18) | **<0.001** |
| Etiology (ref: Cholelithiasis) |  | **0.003** |  | **0.001** |
| Hypertriglyceridemia | 1.31 (0.70–2.43) | 0.397 | 1.70 (0.90–3.22) | 0.105 |
| Alcohol | 2.25 (1.23–4.12) | **0.008** | 2.27 (1.21–4.25) | **0.01** |
| Other | 2.76 (1.49–5.13) | **0.001** | 2.87 (1.49–5.52) | **0.002** |
| Mixed | 1.10 (0.74–1.64) | 0.625 | 0.98 (0.66–1.46) | 0.916 |

Notes: OR = odds ratio; CI = confidence interval. *P* < 0.05 was bolded.

Abbreviations: RAC, revised Atlanta criteria; MCTSI, modified computed tomography severity index; BMI, body mass index; CRP, C-reactive protein; WBC, white blood cell; CREA, creatinine; LDH, lactate dehydrogenase; TC, total cholesterol; TG, triglyceride; HDL, high-density lipoprotein; LDL, low-density lipoprotein; TyG index, triglyceride–glucose index; CTI, C-reactive protein–triglyceride–glucose index.

Supplementary Table 4. Univariate ordinal logistic regression analysis for severity (RAC) and severity (MCTSI)

| **Variable** | **Severity (RAC）** |  | **Severity (MCTSI)** |  |
| --- | --- | --- | --- | --- |
|  | **OR (95% CI)** | ***P* value** | **OR (95% CI)** | ***P* value** |
| Sex (male/female) | 1.576 (1.120–2.218) | **0.009** | 1.611 (1.142–2.272) | **0.006** |
| Age | 0.989 (0.979–0.999) | **0.028** | 0.988 (0.978–0.998) | **0.02** |
| BMI | 0.999 (0.962–1.038) | 0.976 | 1.027 (0.987–1.069) | 0.186 |
| History of use for lipid-lowering medicine (yes/no) | 1.100 (0.719–1.683) | 0.661 | 1.189 (0.771–1.836) | 0.435 |
| Smoking status (yes/no) | 1.623 (1.161–2.273) | **0.005** | 1.899 (1.342–2.688) | **<0.001** |
| Drinking status (yes/no) | 1.718 (1.230–2.398) | **0.001** | 1.863 (1.321–2.626) | **<0.001** |
| Diabetes (yes/no) | 1.174 (0.797–1.727) | 0.417 | 1.292 (0.867–1.926) | 0.207 |
| Hypertension (yes/no) | 0.985 (0.686–1.414) | 0.935 | 0.793 (0.545–1.153) | 0.224 |
| CRP | 1.005 (1.004–1.007) | **<0.001** | 1.007 (1.006–1.009) | **<0.001** |
| WBC | 1.054 (1.020–1.089) | **0.001** | 1.091 (1.053–1.129) | **<0.001** |
| CREA | 1.002 (1.001–1.004) | **0.008** | 1.000 (0.999–1.002) | 0.697 |
| LDH | 1.001 (1.001–1.002) | **<0.001** | 1.001 (1.001–1.002) | **<0.001** |
| Amylase | 1.000 (1.000–1.000) | 0.999 | 0.999 (0.999–1.000) | 0.288 |
| Lipase | 1.000 (1.000–1.000) | 0.386 | 1.000 (1.000–1.000) | 0.122 |
| Calcium | 0.285 (0.139–0.586) | **0.001** | 0.217 (0.102–0.464) | **<0.001** |
| Glucose | 1.041 (1.003–1.080) | **0.034** | 1.079 (1.038–1.122) | **<0.001** |
| TC | 1.053 (1.012–1.097) | **0.012** | 1.089 (1.043–1.137) | **<0.001** |
| TG | 1.012 (1.002–1.021) | **0.014** | 1.022 (1.012–1.033) | **<0.001** |
| HDL | 0.807 (0.615–1.058) | 0.119 | 0.891 (0.696–1.140) | 0.357 |
| LDL | 1.129 (1.011–1.257) | **0.03** | 1.237 (1.103–1.388) | **<0.001** |
| TyG index | 1.231 (1.104–1.376) | **<0.001** | 1.426 (1.266–1.606) | **<0.001** |
| CTI | 1.282 (1.172–1.402) | **<0.001** | 1.476 (1.338–1.628) | **<0.001** |
| High CTI (yes/no) | 2.922 (2.094–4.078) | **<0.001** | 3.824 (2.702–5.412) | **<0.001** |
| Etiology (ref: Cholelithiasis) |  |  |  |  |
| Hypertriglyceridemia | 1.564 (0.859–2.846) | 0.144 | 2.149 (1.156–3.994) | **0.016** |
| Alcohol | 2.175 (1.234–3.835) | **0.007** | 2.494 (1.376–4.520) | **0.003** |
| Other | 2.333 (1.318–4.128) | **0.004** | 3.158 (1.722–5.789) | **<0.001** |
| Mixed | 1.049 (0.713–1.545) | 0.808 | 1.028 (0.694–1.522) | 0.89 |

Notes: OR = odds ratio; CI = confidence interval. *P* < 0.05 was bolded.

Abbreviations: RAC, revised Atlanta criteria; MCTSI, modified computed tomography severity index; BMI, body mass index; CRP, C-reactive protein; WBC, white blood cell; CREA, creatinine; LDH, lactate dehydrogenase; TC, total cholesterol; TG, triglyceride; HDL, high-density lipoprotein; LDL, low-density lipoprotein; TyG index, triglyceride–glucose index; CTI, C-reactive protein–triglyceride–glucose index.

Supplementary Table 5. Univariate logistic regression analysis for severity (RAC) and severity (MCTSI) on raw data

| **Variable** | **Severity（RAC）** |  | **Severity (MCTSI)** |  |
| --- | --- | --- | --- | --- |
|  | **OR (95% CI)** | ***P* value** | **OR (95% CI)** | ***P* value** |
| Sex (male/female) | 1.591 (1.124–2.254) | **0.009** | 1.499 (1.057–2.128) | **0.023** |
| Age | 0.987 (0.977–0.998) | **0.015** | 0.990 (0.980–1.000) | 0.055 |
| BMI | 0.997 (0.960–1.036) | 0.894 | 1.019 (0.979–1.061) | 0.358 |
| History of use for lipid-lowering medicine (yes/no) | 1.142 (0.735–1.770) | 0.556 | 1.116 (0.713–1.748) | 0.631 |
| Smoking status (yes/no) | 1.618 (1.143–2.294) | **0.007** | 1.751 (1.224–2.513) | **0.002** |
| Drinking status (yes/no) | 1.692 (1.198–2.392) | **0.003** | 1.742 (1.220–2.481) | **0.002** |
| Diabetes (yes/no) | 1.149 (0.770–1.715) | 0.495 | 1.403 (0.927–2.123) | 0.11 |
| Hypertension (yes/no) | 0.920 (0.634–1.335) | 0.661 | 0.775 (0.529–1.135) | 0.19 |
| CRP | 1.005 (1.003–1.007) | **<0.001** | 1.009 (1.007–1.012) | **<0.001** |
| WBC | 1.052 (1.016–1.088) | **0.004** | 1.087 (1.047–1.128) | **<0.001** |
| CREA | 1.001 (0.999–1.003) | 0.238 | 1.000 (0.999–1.002) | 0.606 |
| LDH | 1.001 (1.000–1.002) | **0.001** | 1.001 (1.001–1.002) | **0.001** |
| Amylase | 1.000 (1.000–1.000) | 0.909 | 1.000 (1.000–1.000) | 0.568 |
| Lipase | 1.000 (1.000–1.000) | 0.56 | 1.000 (1.000–1.000) | 0.24 |
| Calcium | 0.310 (0.144–0.670) | **0.003** | 0.251 (0.111–0.571) | **0.001** |
| Glucose | 1.033 (0.993–1.074) | 0.106 | 1.082 (1.035–1.131) | **0.001** |
| TC | 1.052 (1.006–1.101) | **0.027** | 1.101 (1.043–1.162) | **<0.001** |
| TG | 1.019 (1.005–1.033) | **0.006** | 1.029 (1.011–1.048) | **0.002** |
| HDL | 0.737 (0.526–1.032) | 0.076 | 0.816 (0.605–1.100) | 0.181 |
| LDL | 1.151 (1.013–1.308) | **0.03** | 1.234 (1.075–1.416) | **0.003** |
| TyG index | 1.225 (1.089–1.377) | **0.001** | 1.401 (1.232–1.592) | **<0.001** |
| CTI | 1.273 (1.158–1.400) | **<0.001** | 1.455 (1.309–1.617) | **<0.001** |
| High CTI (yes/no) | 2.770 (1.972–3.891) | **<0.001** | 3.636 (2.551–5.181) | **<0.001** |
| Etiology (ref: Cholelithiasis) |  | **0.003** |  | **0.001** |
| Hypertriglyceridemia | 1.308 (0.703–2.434) | 0.397 | 1.697 (0.895–3.216) | 0.105 |
| Alcohol | 2.254 (1.234–4.115) | **0.008** | 2.268 (1.211–4.247) | **0.01** |
| Other | 2.759 (1.485–5.127) | **0.001** | 2.865 (1.486–5.524) | **0.002** |
| Mixed | 1.103 (0.744–1.637) | 0.625 | 0.979 (0.657–1.458) | 0.916 |

Notes: OR = odds ratio; CI = confidence interval. *P* < 0.05 was bolded.

Abbreviations: RAC, revised Atlanta criteria; MCTSI, modified computed tomography severity index; BMI, body mass index; CRP, C-reactive protein; WBC, white blood cell; CREA, creatinine; LDH, lactate dehydrogenase; TC, total cholesterol; TG, triglyceride; HDL, high-density lipoprotein; LDL, low-density lipoprotein; TyG index, triglyceride–glucose index; CTI, C-reactive protein–triglyceride–glucose index.

Supplementary Table 6. Sensitivity analysis comparing MI and CCA in multivariable models for severity of AP

| **Variable** | **Analysis method** | **Severity (RAC)** |  | **Severity (MCTSI)** |  |
| --- | --- | --- | --- | --- | --- |
|  |  | **OR (95% CI)** | ***P* value** | **OR (95% CI)** | ***P* value** |
| CTI | MI (Primary) | 1.180 (1.027–1.357) | **0.019** | 1.436 (1.237–1.667) | **<0.001** |
|  | CCA (Sensitivity) | 1.170 (1.019–1.343) | **0.026** | 1.426 (1.227–1.658) | **<0.001** |
| WBC | MI (Primary) | 1.025 (0.988–1.064) | 0.18 | 1.050 (1.009–1.092) | **0.015** |
|  | CCA (Sensitivity) | 1.018 (0.981–1.057) | 0.351 | 1.042 (1.001–1.085) | **0.043** |
| LDH | MI (Primary) | 1.001 (1.000–1.002) | **0.009** | 1.001 (1.000–1.002) | **0.048** |
|  | CCA (Sensitivity) | 1.001 (1.000–1.002) | **0.014** | 1.001 (1.000–1.002) | 0.067 |

Notes: MI = multiple imputation; CCA = complete case analysis; OR = odds ratio; CI = confidence interval. Primary analysis refers to the main multivariable models using MI (20 imputed datasets); Sensitivity analysis refers to the same models using CCA to assess robustness to missing data. *P* < 0.05 was bolded.

Abbreviations: AP, acute pancreatitis; RAC, revised Atlanta criteria; MCTSI, modified computed tomography severity index; CTI, C-reactive protein–triglyceride–glucose index; LDH, lactate dehydrogenase; WBC, white blood cell.

Supplementary Table 7. Sensitivity analysis for the association between CTI and AP severity with additional adjustment for BMI

| Severity Classification | Model | **CTI OR (95% CI)** | ***P* value** | **BMI OR (95% CI)** | ***P* value** |
| --- | --- | --- | --- | --- | --- |
| RAC-defined | Primary (without BMI) | 1.180 (1.027–1.357) | **0.019** | — | — |
|  | Sensitivity (with BMI) | 1.252 (1.082–1.448) | **0.003** | 0.932 (0.889–0.977) | **0.004** |
| MCTSI-defined | Primary (without BMI) | 1.436 (1.237–1.667) | **<0.001** | — | — |
|  | Sensitivity (with BMI) | 1.513 (1.291–1.774) | **<0.001** | 0.946 (0.899–0.994) | **0.029** |

Notes: OR = odds ratio; CI = confidence interval. Sensitivity models were adjusted for BMI in addition to all covariates included in the primary models (sex, age, smoking, drinking, WBC, LDH, calcium, and etiology). Primary model results are reproduced from Table 3 for direct comparison. *P* < 0.05 was bolded.

Abbreviations: CTI, C-reactive protein–triglyceride–glucose index; AP, acute pancreatitis; BMI, body mass index; RAC, revised Atlanta criteria; MCTSI, modified computed tomography severity index; WBC, white blood cell; LDH, lactate dehydrogenase.
